# Nano-based drug delivery systems for active ingredients from traditional Chinese medicine: Harnessing the power of nanotechnology

**DOI:** 10.3389/fphar.2024.1405252

**Published:** 2024-06-07

**Authors:** Yong-Bo Zhang, Jun-Fang Wang, Mei-Xia Wang, Jing Peng, Xiang-De Kong, Jie Tian

**Affiliations:** Department of Pharmacy, Affiliated Hospital of Jining Medical University, Jining, China

**Keywords:** TCM, active ingredients, nanotechnology, nano-TCM, drug delivery

## Abstract

**Introduction:** Traditional Chinese medicine (TCM) is gaining worldwide popularity as a complementary and alternative medicine. The isolation and characterization of active ingredients from TCM has become optional strategies for drug development. In order to overcome the inherent limitations of these natural products such as poor water solubility and low bioavailability, the combination of nanotechnology with TCM has been explored. Taking advantage of the benefits offered by the nanoscale, various drug delivery systems have been designed to enhance the efficacy of TCM in the treatment and prevention of diseases.

**Methods:** The manuscript aims to present years of research dedicated to the application of nanotechnology in the field of TCM.

**Results:** The manuscript discusses the formulation, characteristics and therapeutic effects of nano-TCM. Additionally, the formation of carrier-free nanomedicines through self-assembly between active ingredients of TCM is summarized. Finally, the paper discusses the safety behind the application of nano-TCM and proposes potential research directions.

**Discussion:** Despite some achievements, the safety of nano-TCM still need special attention. Furthermore, exploring the substance basis of TCM formulas from the perspective of nanotechnology may provide direction for elucidating the scientific intension of TCM formulas.

## Graphical Abstract

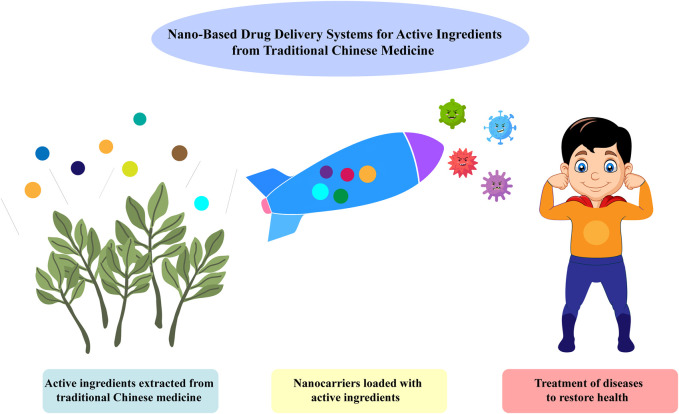

## 1 Introduction

Since ancient times, plants have been widely used as medicinal agents for various diseases. TCM, which consists of plants, animals, and minerals, has been used in China for thousands of years (Wang et al., 2018). Among the various species used in TCM, plants account for 90% (Zuo et al., 2021). Plants are rich in bioactive metabolites, which offers the potential to treat a wide range of diseases. Based on the inherent advantages of natural products, active ingredients of TCM are currently being screened for the treatment of diseases such as cancer, diabetes, cardiovascular diseases and inflammation. For example, it has been found that flavonoids and non-flavonoid polyphenolic compounds exhibit favorable anti-inflammatory effects both *in vivo* and *in vitro* (Peng et al., 2023). The alkaloid berberine (BBR) extracted from *Coptis chinensis* Franch. has anti-inflammatory effect. Camptothecin (CPT) is extracted from *Camptotheca acuminata* Decne., which has a good anti-tumor effect (Swamy et al., 2021; Lan et al., 2022).

However, the compositional complexity and toxicity-related issues associated with herbal ingredients pose greater challenges to their use as medicines and therefore make it difficult to transition from clinical trial to the use of nanotechnology (Patra et al., 2018; Peng et al., 2023). Using drug delivery systems to deliver these ingredients may be an option to address these issues. Nanotechnology has been introduced into the research of TCM, leading to the concept of nano-TCM. By incorporating natural products in nanocarriers, their properties, such as bioavailability, targeting ability and controlled release, can be effectively improved. In nano-based delivery of herbal ingredients, organic, inorganic, and polymeric nanostructures, including nanoparticles (NPs), micelles, liposomes, and dendrimers, are often considered (Patra et al., 2018).

Nanotechnology has been widely applied in various aspects of TCM (Zheng et al., 2022). Herein, this review focused on the integration of nanotechnology with natural products derived from Chinese herbs. Different drug delivery systems based on TCM have been carefully designed and can be classified into two platforms: nanocarriers and carrier-free nanomedicines. Liposomes, micelles, NPs, and dendrimers are commonly used nanocarriers. Carrier-free nanomedicines include self-assembled nanomedicines, pharmaceutical cocrystals, and Pickering emulsions. Despite these achievements, further understanding of the safety of nano-TCM is necessary to accelerate future clinical translation. Moreover, exploring how TCM theories, such as personalized diagnosis and prescription, can be better incorporated into modern research requires further investigation. Overall, the remarkable progress in nano-TCM highlights the significant role of nanotechnology in advancing the modernization of TCM research.

## 2 Active ingredients of TCM

**FIGURE 1:**
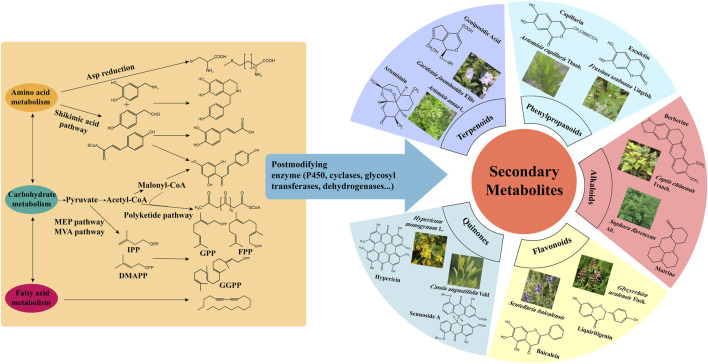
Synthesis of active ingredients of TCM.

TCM is a large repository of bioactive metabolites including terpenes, flavonoids, alkaloids, glycosides, saponins and so on. For example, ginkgolides are diterpenes, ginsenosides are triterpenes, baicalin is a flavonoid, and BBR is a benzylisoquinoline alkaloid. According to statistics, 25% of new molecular entities approved by the U.S. Food and Drug Administration (FDA) from natural sources are derived from plant natural products (Li and Weng, 2017). As shown in Figure 1, medicinal plants produce structurally and functionally diverse secondary metabolites. These compounds serve as the material basis for the therapeutic effects of TCM and are also sources for innovative drugs.

## 3 Nano-TCM

Based on the clinical validation of several active ingredients, such as artemisinin (ART), paclitaxel (PTX), and BBR, TCM is being increasing accepted as a potential source of clinical drugs. However, due to strong hydrophobicity, poor *in vivo* stability, low bioavailability, and potential systemic toxicity within therapeutic doses, some ingredients fail to meet the requirements for clinical application (Watkins et al., 2015). Nano-TCM offers several advantages over herbal medicines. It improves bioavailability, enables sustained release, and enhances the solubility and permeability of poorly soluble drugs, allowing them to overcome biological barriers (Watkins et al., 2015). The application strategies for nano-TCM can be broadly categorized into two types. The first type is nanocarriers, which focus on the “efficacy” of TCM’s ingredients. It involves the development of novel carriers, such as liposomes, NPs, dendrimers, and micelles to encapsulate and deliver these active ingredients. The second type is carrier-free nanomedicine, which emphasizes the “functionality” of certain herbal ingredients. In this approach, these ingredients act as solubilizers, stabilizers, or targeting ligands, exerting therapeutic effects while also serving as carriers themselves.

### 3.1 Advantages of nano-TCM

#### 3.1.1 Improved bioavailability

Many identified ingredients of TCM, such as curcumin (CUR), resveratrol (RES), and ART, are lipophilic in nature. Due to their poor solubility in the bloodstream, these compounds often require high doses for therapeutic efficacy, which can lead to toxicity or poor patient compliance (Muqbil et al., 2011). On the other hand, alkaloids such as ephedrine (EPH) and matrine (MT) are hydrophilic and encounter obstacles in crossing biological membranes (Bonifácio et al., 2014). Encapsulating these active ingredients in nanocarriers can enhance their bioavailability and reduce the required dosage to achieve therapeutic effects. CUR is a polyphenolic compound derived from the rhizome of *Curcuma longa* Linn., and it possesses anti-inflammatory, antioxidant, and anticancer properties (Kadota et al., 2020). Takahashi et al. (2009) encapsulated CUR in lipid-based nanocarriers (LECs). The area under the curve (AUC) analysis revealed that the AUC value in rats after oral administration of LECs was 4.96 times greater than that of free CUR. In another study, the relative bioavailability of CUR-loaded lipid polymeric NPs was increased 18.2-fold compared to free CUR (Liu Y. et al., 2019).

#### 3.1.2 Targeted delivery

The second advantage of nano-TCM is its ability to target specific tissues or organs. Targeted delivery can increase the proportion of drugs reaching specific tissues to improve bioavailability and reduce drug side effects. Targeted delivery strategies can be divided into two categories: active targeting, which involves attaching targeting ligands to the surface of the carrier, and passive targeting, which relies on inherent properties such as size, shape, and surface charge to reach the target area without specific chemical interactions.

Active targeting is achieved by attaching different types of ligands, such as peptides, antibodies, proteins, and nucleic acids, to the surface of the carrier to improve the target to non-target ratio. Nanocarriers conjugated with folic acid (FA) have shown promise in cancer treatment. Due to the overexpression of folic receptors, FA-grafted nanocarriers can target cancer cells. Hong et al. (2021) synthesized β-cyclodextrin-polycaprolactone block copolymers and conjugated them with FA to construct CUR-loaded nanoparticles (FA-CUR-NPs) using the emulsion evaporation method. Under tumor microenvironment conditions (pH 6.4), the release rate of CUR from FA-CUR-NPs was three times greater than that under systemic circulation conditions (pH 7.4). Compared to free CUR and CUR-NPs, oral administration of FA-CUR-NPs reduced the tumor volume by three times and two times, respectively, after 30 days in mice. These indicates that FA-CUR-NPs demonstrate significantly improved therapeutic efficacy *in vivo* and that FA can be successfully used as a tumor-targeting ligand to enhance cellular internalization.

Lactoferrin (LF) is a glycoprotein of the transferrin (TF) family that can bind to TF receptors (TFRs) and LF membrane internalization receptors (LFRs) that are highly expressed on the surface of cancer cells and blood-brain barrier (BBB), thereby promoting entry into the cell nucleus (Agwa and Sabra, 2021). This characteristic can be used to develop active targeted drug delivery systems. A novel mesoporous magnetic nanocarrier was formed by grafting LF onto mesoporous oxide nanoparticles (MIONs) through an EDC coupling reaction. The nanocarrier can continuously release perfluorohexane (PFH) and PTX to achieve deep penetration of drugs in tumors. Prior to exposure to high-frequency magnetic fields (MF), LF-MIONs loaded with PTX and PFH exhibited slow *in vitro* release. A local increase in the temperature of the MIONs triggered the vaporization of PFH, leading to severe damage to the tumor spheroids. Additionally, it promoted deep penetration and increased accumulation of the nanocarrier within the tumor, thus increasing the killing potential. After a single exposure to a magnetic field for 16 days, significant inhibition of tumor growth was observed (Su et al., 2015).

Passive targeting is often an effective and cost-effective choice. Many tumors exhibit enhanced permeability and retention (EPR) effects due to vascular leakage, which is the main driving force for passive targeting (Maeda et al., 2013). NPs utilize the EPR effect to deliver drug molecules to the tumor site in a controlled and targeted manner, demonstrating significant advantages in cancer treatment. Chen et al. (2023) prepared pH-responsive and biodegradable calcium orthophosphate@liposomes (CaP@Lip) NPs for loading hydrophobic PTX and hydrophilic doxorubicin (DOX) hydrochloride. Under physiological conditions, the obtained NPs carry a negative charge but they convert to a positive charge when exposed to a weakly acidic environment, thereby promoting drug internalization. Additionally, CaP@Lip NPs undergo degradation under acidic conditions (pH 5.5), facilitating drug release and rapid metabolism of the NPs in the body. At pH 5.5, nearly 63.33% of PTX was released within 48 h, while at pH 7.4, the 40.47% of the PTX was released. The slow release of PTX in NPs and the pH-responsive drug release minimize the adverse effects on healthy cells, making it beneficial for tumor treatment (Chen et al., 2023).

#### 3.1.3 Controlled release

The third advantage of nano-TCM is the ability to control drug release. The amount and rate of drug release from nanocarriers depend on various factors, such as the carrier material, formulation size, drug molecules, and microenvironment (Watkins et al., 2015). The choice of carrier material significantly influences drug release characteristics, and the type of polymer can be adjusted to affect the release profile. By polymerizing folic acid-conjugated nanocellulose (FA-NC) with glycidyl methacrylate (GMA) and 2-hydroxyethyl methacrylate (HEMA), Anirudhan et al. prepared an innovative drug delivery system. The hydrogen bonding interaction between the polymer carrier and CUR enhanced the loading efficiency of CUR. Approximately 91.0% of the drug was released within 48 h under acidic conditions, demonstrating controlled release without premature leakage (Anirudhan et al., 2021). In another study, Hu et al. (2023) used a zinc-based nanoscale metal-organic framework (NMOF) as carriers to prepare BR@Zn-BTB NPs loaded with BBR. It was further encapsulated using a hydrogel with ROS scavenging ability (due to the introduction of quaternary ammonium and phenyl borate functionalities) to obtain a BR@Zn-BTB gel (BZ-Gel). As the pH increased, the drug release rate from the BZ-Gel also increased. At pH 8.0, the 36-h release rate of BR was close to 80%, while at pH 7.4, it was approximately 60%, and at pH 7.0 and 6.5, it was close to 40%. In chronic and infectious wounds, the pH of the skin surface becomes alkaline (between 7.5 and 8.9) (Zhu et al., 2020). Therefore, this pH-responsive drug release BR-Gel is suitable for treating malignant diabetic foot wounds. In addition to pH responsiveness, controllable drug release nanocarriers also include temperature-sensitive (Qi X.-J. et al., 2020), redox-responsive (Guo et al., 2017), ion-sensitive (Li et al., 2015) and other types. Taking into account the target location, properties of natural compounds, and preferred carrier materials, optimizing nanocarriers will yield significant benefits.

### 3.2 Nanocarriers

**FIGURE 2:**
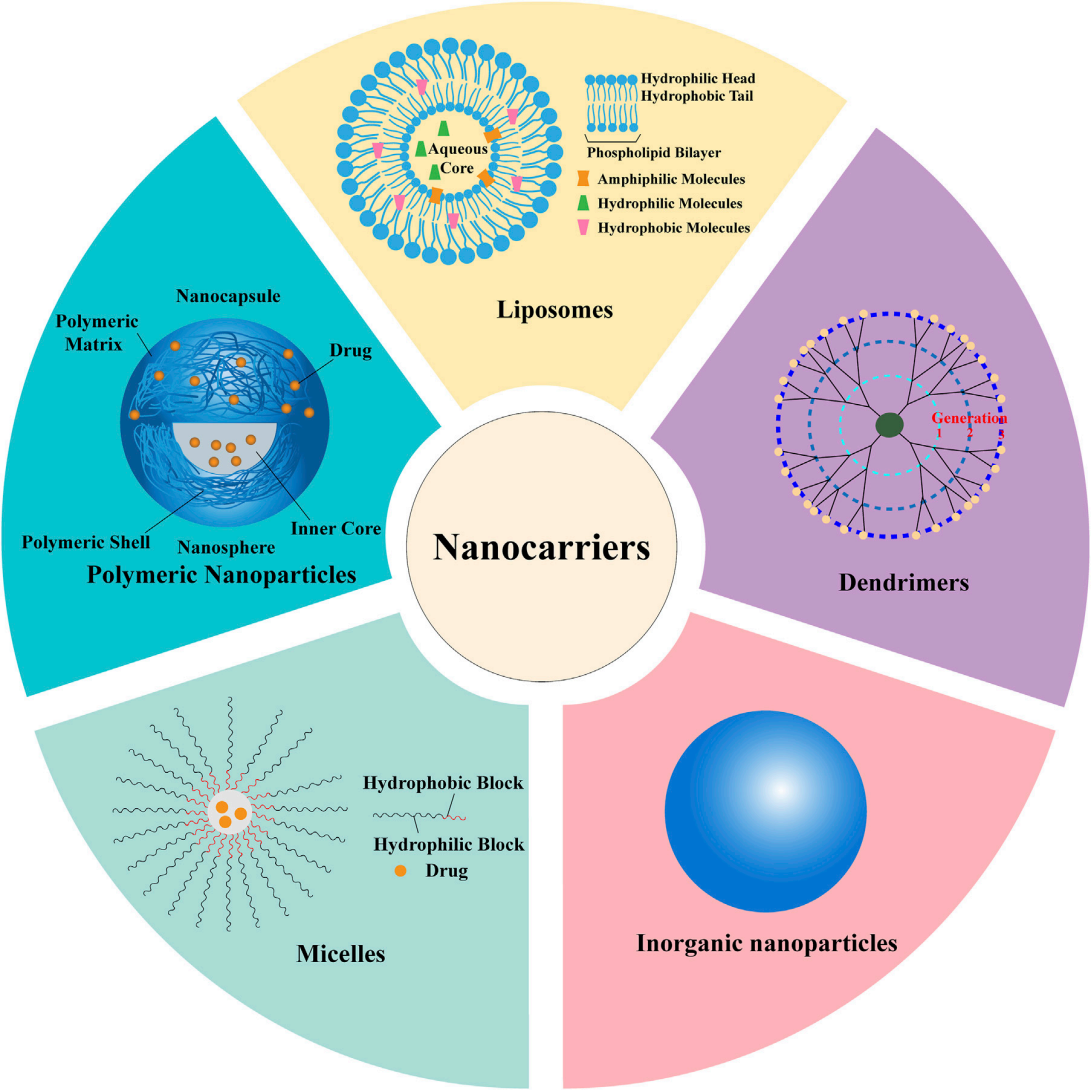
Nanocarriers for delivering active ingredients of TCM.

Developing single herbal ingredient into nanomedicines is considered an innovative strategy in the development of new drugs. The strategy of nanocrystallization of single active ingredients is similar to existing mature chemical drug preparation methods, making it technically feasible. This approach allows the active ingredients to fully exert their efficacy while avoiding the challenges of the complex material basis and quality control of TCM. In recent years, researchers have extensively explored various nanocarriers, including NPs, liposomes, micelles, dendrimers, and so on (Figure 2). These new formulations help overcome the low water solubility and bioavailability issues of herbal ingredients, enabling targeted delivery to specific sites or prolonging circulation in the body.

#### 3.2.1 Liposomes

Liposomes, a commonly used delivery systems for natural products, were first discovered by Bangham et al. in the 1960s, with the first publication in 1964 (Bangham and Horne, 1964). Liposomes are spherical structures composed of lipid molecules with both hydrophilic and hydrophobic properties (Sebaaly et al., 2016). Driven by hydrophobic interactions and other molecular interactions, amphiphilic lipid molecules spontaneously assemble into liposomes in an aqueous environment. The liposome membrane can consist of one or multiple lipid bilayers. With an aqueous core inside, the polar head groups face the inner and outer aqueous phases (Manna et al., 2019). This structure endows liposomes with the ability to encapsulate molecules with different solubilities. Lipophilic drugs can be encapsulated within phospholipid bilayers or adsorbed onto the surface of liposomes, while hydrophilic drugs can be encapsulated by the internal aqueous core. Additionally, liposomes exhibit excellent biocompatibility and biodegradability due to their phospholipid bilayer structure, facilitating favorable interactions with cell membranes and promoting effective cellular uptake (He et al., 2019).

##### 3.2.1.1 Preparation of liposomes

The preparation techniques for liposomes can be divided into traditional methods and novel methods, and different techniques can have an impact on the final characteristics of liposomes, such as size and encapsulation efficiency (EE). Traditional methods include film hydration, reverse-phase evaporation, solvent injection, and detergent removal. These methods generally involve four steps: 1) dissolution of lipids (usually using organic solvents), 2) removal of organic solvents, 3) purification and separation of liposomes, and 4) analysis of the final liposomes (Akbarzadeh et al., 2013).

The film hydration method, also known as the Bangham method, was the earliest reported technique for liposome preparation (Bangham et al., 1967). In this method, lipids are generally dissolved in organic solvents such as ether, chloroform, or methanol, and then a lipid film is formed by evaporating and drying the organic solvent. The lipid film is then hydrated using an aqueous solvent to form liposomes (Nkanga et al., 2019). The main disadvantages of this method are the production of large and uneven liposomes, low EE, and difficulty in completely removing organic solvents.

The initial steps of the reverse-phase evaporation method are similar to those of the film hydration method. First, phospholipids are dissolved in an organic solvent to form a thin film, after which the solvent is evaporated to remove them. Next, the film is redissolved in an organic solvent, and water is added to form a water-in-oil emulsion (Pattni et al., 2015). The emulsion was then subjected to ultrasound treatment to make it more uniform. Finally, the organic solvent is evaporated under reduced pressure to form a liposome suspension (Akbarzadeh et al., 2013). The advantage of this method is that it achieves high EE in liposomes, but the disadvantage is that the encapsulated compounds are exposed to ultrasound conditions and organic solvents (Monteiro et al., 2014).

The solvent injection method involves rapidly injecting a lipid solution dissolved in an organic solvent into an aqueous medium to form liposomes (William et al., 2020). This method is commonly used for liposome preparation due to its simplicity, strong reproducibility, fast speed, and minimal lipid degradation or oxidation. However, this method still has several limitations, such as poor solubility of certain compounds in ethanol, low EE for hydrophilic compounds, and incomplete removal of ethanol (Maherani et al., 2011).

In the detergent removal method, phospholipids are dissolved by a detergent at the critical micelle concentration (Nkanga et al., 2019). After removing the detergent by column chromatography or dialysis, the phospholipid molecules self-assemble in an aqueous medium to form liposomes (Akbarzadeh et al., 2013). The size and uniformity of the liposomes produced by this method can be influenced by the initial ratio of phospholipids to detergents and the efficiency of detergent removal (Maherani et al., 2011). The disadvantage of this method is that impurities may be present in liposomes, and interactions between the detergent and the compounds can also occur.

Currently, research on new liposome preparation methods has focuses mainly on expanding the industrial production scale and making them suitable for various phospholipids and drugs (Pattni et al., 2015). Some of these new methods are improvements on traditional methods, such as direct hydration of lipid components after ultrasound treatment to avoid dissipation (Manca et al., 2013). Additionally, the application of supercritical fluid (SCF) technology in liposome production has also been explored. This method utilizes a supercritical fluid, such as CO_2_, maintained under supercritical conditions. The SCF method offers several advantages, including low solvent cost, environmental friendliness, controllable particle size, *in-situ* sterilization, and suitability for large-scale production (William et al., 2020).

##### 3.2.1.2 Application of liposomes

**FIGURE 3:**
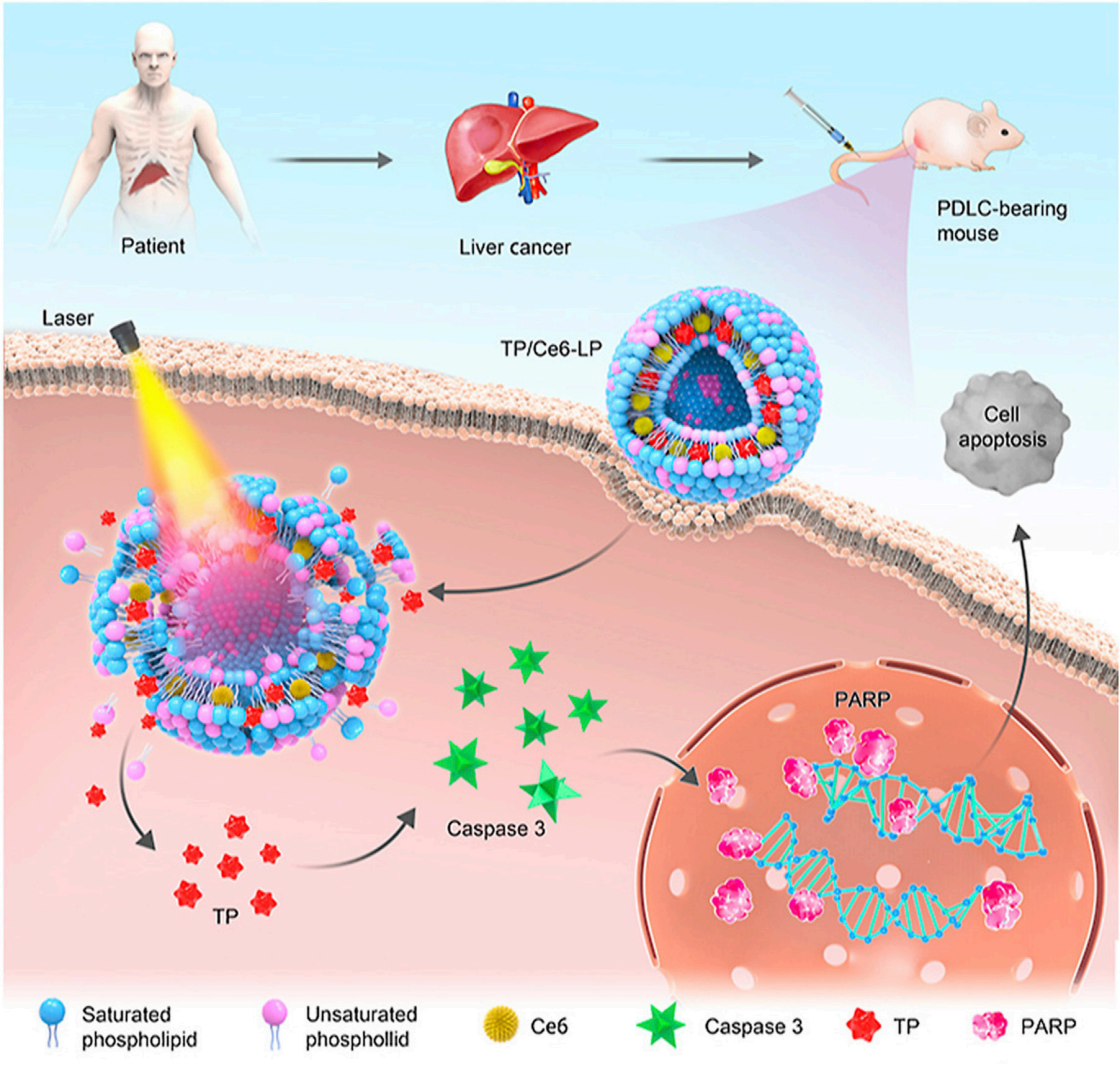
Schematic illustration of preparation, targeting mechanism, synchronous intracellular drug release, and visualization of photosensitive liposome@TP (TP/Ce6-LP). Reprinted from Acta Pharmaceutica Sinica B, L. Yu, Z. Wang, Z. Mo, B. Zou, Y. Yang, R. Sun, W. Ma, M. Yu, S. Zhang, Z. Yu, Synergetic delivery of triptolide and Ce6 with light-activatable liposomes for efficient hepatocellular carcinoma therapy, 2004-2015, Copyright (2021), with permission from Elsevier [OR APPLICABLE SOCIETY COPYRIGHT OWNER].

Triptolide (TP) is an epoxy diterpenoid compound isolated from *Tripterygium wilfordii* Hook F. that has demonstrated anti-inflammatory, anti-tumor, and anti-infective properties (Chen et al., 2018). However, the narrow therapeutic window, poor water solubility, and rapid metabolism of TP limit its clinical application. To reduce adverse reactions and improve treatment efficacy, Yu et al. (2021) designed a light-activated liposome (TP/Ce6-lp). By combining the photosensitizer Ce6 with TP, this liposome can synergistically treat liver cancer through the controlled release of TP and photodynamic therapy. Studies on its anti-tumor activity have shown that TP/Ce6-lp induces cell apoptosis by upregulating Caspase-3/PARP protein expression, resulting in good therapeutic effects on patient-derived hepatocellular carcinoma xenografts (PDX^HCC^) after irradiation (Figure 3).

In the field of TCM, TP is commonly used to treat rheumatoid arthritis. To improve the transdermal delivery of TP in collagen-induced arthritis (CIA) rats, Chen et al., 2015) prepared a TP-loaded liposome hydrogel (TP-LHP) in the form of a microneedle patch and evaluated its pharmacokinetics and pharmacodynamics. The results showed that, after 1 week of treatment, TP-LHP had the effect on reducing joint swelling in all treatment dose groups, with the high-dose group showing the greatest efficacy. TP-LHP demonstrated sustained and stable release of TP, and significant efficacy was observed after 4 weeks of continuous treatment, indicating that the combination of TP-LHP and the microneedle delivery strategy is effective for the treatment of rheumatoid arthritis.

The active ingredients of TCM can also participate in the construction of liposomes, which can exert dual effects. In recent years, numerous published reports have shown that combination therapy with TCM can effectively improve tumor conditions and have synergistic effects with reduced toxicity (Chen et al., 2021; Lu et al., 2021). To overcome the potential toxic effects of traditional liposome formulations in the body (Moein Moghimi et al., 2006), Guo et al. (2022) incorporated glycyrrhizic acid (GA) into liposomes constructed with a mixture of saponins and phospholipids, using platycodin and ginsenoside as substitutes for cholesterol to construct saponin liposomes (RP-lipo). PR-lipo@GA exhibited similar morphological characteristics and drug release behavior to conventional liposomes but demonstrated stronger lung cancer cell targeting and anti-tumor capabilities *in vitro*, possibly attributed to the pharmacological properties of saponins themselves (Lu et al., 2018). This novel formulation of liposomal drug delivery system not only challenges the status of cholesterol as a component of liposomes but also provides an innovative system for the clinical application of combination therapy.

#### 3.2.2 Polymeric micelles

##### 3.2.2.1 Preparation of polymeric micelles

Polymeric micelles are core-shell aggregates formed by self-assembly of amphiphilic block copolymers at the critical micelle concentration. Depending on the hydrophobic and hydrophilic conditions as well as the solvent, micelles can adopt various shapes, including spherical, cylindrical, inverse micellar, and bottle-brush structures. The preparation methods for micelles include dilution (Liu et al., 2006), freeze-drying (Teagarden and Baker, 2002), solvent evaporation (Hibino et al., 2021), and dialysis (Minatti et al., 2003). During the preparation process, the physicochemical properties of the block copolymers, the sequence of addition, concentration, and water/organic solvent ratio can impact the size, polydispersity index, and stability of the micelles (Kotta et al., 2022). Micelles are capable of loading hydrophobic drugs into their core through physical encapsulation, chemical conjugation, and electrostatic interactions, exhibiting excellent stability and drug solubility in aqueous environments. Table 1 summarizes the materials and preparation methods of polymeric micelles used for loading components of Chinese herbs.

**TABLE 1:** Study of polymeric micelles loaded with active ingredients of TCM.

Polymer	Method of preparation	Drug	Ref.
Hexyl-hyaluronan and oleyl-hyaluronan	Solvent evaporation method	Curcumin	Starigazdová et al. (2020)
Block copolymer of mPEG (5 kDa)-PCL (2 kDa)	Continuous processing	Curcumin	Gupta et al. (2020)
disteraroylphosphatidylethanolamine-PEG	Solvent evaporation method	Paclitaxel	Oda et al. (2020)
Carboxymethyl chitosan-rhein conjugate	Self-assembly	Paclitaxel	Wang et al. (2020a)
Gallic acid-Chitosan-D-α-tocopherol PEG 1000 succinate	Ultrasonic emulsification	Paclitaxel	Tu et al. (2020)
Cholic acid conjugated poly (bis (carboxyphenoxy) phosphazene)	Ultrasonication	Paclitaxel	Mehnath et al. (2020)
Chitosan-lecithin	Sonication	Thymoquinone	Negi et al. (2020)

##### 3.2.2.2 Application of polymeric micelles

Polymeric micelles, as drug carriers, have nanoscale sizes and narrow size distributions. The core-shell structure of these materials helps shield drugs from oxidation, enhancing drug stability. PTX, a type of diterpenoid alkaloid compound, has been found to induce cell cycle arrest and apoptosis in tumor cells by polymerizing tubulin dimers to stabilize microtubules (Bian et al., 2015). However, the anti-tumor potential of PTX is hindered by its poor water solubility, short biological half-life, and toxicity to normal tissues. Encapsulating PTX in biocompatible carriers is an alternative approach for targeted drug delivery. Wang Y. et al. (2020) synthesized a biotin-functionalized block copolymer, which called poly (*N*-2-hydroxypropylmethacrylamide)-block-poly (*N*-2-benzoyloxypropyl methacrylamide). This copolymer can self-assemble into polymer micelles in water, and its size is positively correlated with the length of hydrophobic segments. Due to the presence of biotin receptors on the surface of target cells, biotin-modified micelles achieve more effective internalization and exert stronger cytotoxicity. In another study, Huang et al. (2018) prepared a pH-responsive prodrug of PTX, which consisted of amphiphilic polyethylene glycol (PEG) and PTX. In the acidic environment of tumor tissues, the aldehyde linker is cleaved, resulting in the rapid release of PTX loaded in the micelles, followed by the release of conjugated PTX, thereby achieving programmable drug release.

Studies have shown that CUR inhibits tumor generation, proliferation, and metastasis by downregulating cyclin B1, activating the caspase-9/3 cascade, inhibiting the PI3K/Akt/mTOR signaling pathway, and suppressing matrix metalloproteinase-2 (MMP-2) (Yang et al., 2014). To overcome the poor water solubility of CUR, Sun et al. (2021) synthesized galactosamine-modified polyethylene glycol-polylactic acid (Gal-PEG-PLA) polymers, and prepared CUR-loaded Gal-PEG-PLA/D-α-tocopherol polyethylene glycol 1,000 succinate micelles (CUR-loaded Gal-PEG-PLA/TPGS). The size of these polymeric micelles is approximately 100 nm, with a drug loading capacity of 14.6%. The biodistribution results showed significant absorption of these micelles in the jejunum and ileum. Moreover, CUR-loaded micelles can reduce damage to liver and intestinal tissues, making them valuable for the oral administration of hydrophobic drugs.

#### 3.2.3 NPs

NPs are a novel drug delivery system that are actually defined as “solid colloidal particles”. The particle size generally ranges from 10–50 nm, with an upper limit of about 1,000 nm (Mora-Huertas et al., 2010; Petros and DeSimone, 2010). NPs can load a wide range of drugs, including proteins, hydrophobic drugs, hydrophilic drugs, vaccines, and biomacromolecules. Through formulation design, NPs can achieve targeted drug delivery to organs such as the lymphatic system, spleen, lungs, brain, and liver, and can prolong the circulation time of drugs in the body (Anwar et al., 2021). NPs provide an ideal choice for the controlled and targeted administration of natural products and have attracted great interest from researchers.

##### 3.2.3.1 Polymeric NPs

Polymeric NPs are colloidal systems composed of natural, synthetic, or semi-synthetic polymers (Van Vlerken et al., 2007). Compared to inorganic NPs, polymeric NPs typically exhibit good biocompatibility, stability, processability, and responsiveness to external stimuli (Sarcan et al., 2018). The polymer serves as the backbone of polymeric NPs and is considered the foundation of their composition. Therefore, researchers must understand the characteristics of polymers, such as biocompatibility, biodegradability, stability, permeability, and the interaction between drugs and polymers, in order to select appropriate formulations. Additionally, the properties of the formulation system can be modulated through chemical modifications, the addition of targeting molecules, the incorporation of lipids, and other methods to achieve the desired objectives of the researchers (Kumari et al., 2010).

Polymers can be classified into natural polymers and synthetic polymers based on their source. Numerous studies have reported the use of synthetic polymers for the preparation of polymeric NPs. Commonly used synthetic polymers include polylactic acid (PLA), poly (lactic-co-glycolic acid) (PLGA), and poly-ε-caprolactone (PCL). For example, Rathinavel et al. (2021) prepared polymeric NPs loaded with CUR using PCL to enhance the antibacterial effects against both gram-positive and gram-negative strains. Thuy et al. (Kang et al., 2022) used PAMAM to prepare co-loaded NPs of PTX and CUR, achieving improved bioavailability and enhanced anticancer activity against skin cancer. Kumar et al. (2014) prepared methacrylate-based NPs for CUR delivery and observed that the drug delivery system enhanced the antitumor activity and significantly reduced G0/G1 cell cycle arrest in tumor cells.

Compared to synthetic polymers, natural polymers have gained widepraed attention due to their high biocompatibility, biodegradability, stability, and cost-effectiveness (Sabra et al., 2019). Commonly used natural polymers include chitosan, alginate, and gelatin. Chitosan is a cationic alkaline polysaccharide that exhibits good biocompatibility and biodegradability and contains a large number of functional groups suitable for chemical modification. Methods for preparing chitosan NPs include ion gelation (Kalpana et al., 2010), microemulsion (Wang et al., 2008), and emulsion solvent diffusion (El-Shabouri, 2002). Rahmati et al. (2021) studied the preparation of BBR-loaded alginate/chitosan gel and evaluated its therapeutic efficacy in a rat sciatic nerve crush injury model. MTT assays confirmed the cell compatibility of the gel and demonstrated the dose-dependent effect of BBR on cell proliferation. *In vivo* experiments showed that the hydrogel containing 1% BBR had a positive effect on rat sciatic nerve regeneration. Dogan investigated the potential cytotoxic effects of quercetin (QUE) and QUE-loaded chitosan NPs on SH-SY5Y cells. After treatment with different concentrations of QUE (0.5, 1, 2, 4, 8 μg/mL) for 24 h, cell viability was determined using the XTT assay. The results showed that QUE-loaded chitosan NPs induced significant cytotoxicity in SH-SY5Y cells through the generation of oxidative stress and DNA damage (Dogan, 2022).

Alginate is an anionic water-soluble natural polymer with biodegradable, biocompatible, and adhesive properties. Its adhesiveness is mainly attributed to strong hydrogen bonding formed through hydroxyl and carboxyl groups interacting with adhesive glycoproteins (Nair and Laurencin, 2007). Methods for preparing alginate NPs include ion gelation, covalent cross-linking, emulsion solvent displacement, and emulsion-solvent evaporation. Ion gelation is a widely used technique for preparing alginate NPs(Damelin et al., 2015), as alginate has an affinity for multivalent cations such as Ca^2+^ and Zn^2+^(Draget et al., 1997). Ahmady et al. (2023) developed a drug delivery system based on alginate. First, alginate NPs loaded with capsaicin were prepared using cationic surfactants and nano-emulsions. The particle size of these NPs was 19.42 ± 11.8 nm, with an encapsulation efficiency of 98.7% ± 0.6%. Subsequently, poly (ε-caprolactone)-chitosan co-blended nanofibers with different mixing ratios were fabricated using electrospinning. The nanofibers with the most favorable characterization results were chosen to encapsulate the alginate NPs, resulting in a drug delivery system consisting of nanoparticle-nanofiber composites. *In vitro* analysis demonstrated the effective inhibition of MCF-7 human breast cancer cell proliferation by the designed nanoplatform, while it exhibited no toxicity toward human dermal fibroblasts (HDF).

Gelatin is a protein obtained by partially hydrolyzing collagen to convert it into a non-oriented protein. Based on the pH at which collagen is hydrolyzed, gelatin can be divided into two types: gelatin A (isoelectric point of 9), where collagen is hydrolyzed under acidic conditions, and gelatin B (isoelectric point of 5), where collagen is hydrolyzed under alkaline conditions. Since gelatin is water-soluble, cross-linking of gelatin may be required in the development of NPs(Lin et al., 2009; Elmowafy et al., 2023). Tumor-responsive nanocarriers are highly valuable and in demand for smart anticancer drug delivery. For this reason, Zhou et al. (2020) designed redox- and MMP-2-sensitive NPs for the delivery of PTX. Bovine serum albumin was used as the targeting ligand, and the disulfide-containing prodrug (PTX-SS-COOH) was grafted onto sulfhydryl-modified gelatin as the hydrophilic carrier. The sulfhydryl groups on gelatin can self-cross-link in air to form disulfide bonds, thus giving the NPs a stable structure. Because of their sensitivty to changes in MMP-2 concentration and redox potential, the NPs achieved multi-responsive drug delivery to the tumor microenvironment and showed excellent anti-cancer efficacy in further *in vitro* and *in vivo* experiments.

##### 3.2.3.2 Inorganic NPs

The nanoscale synthesis of inorganic materials has led to significant changes in biology and medicine. With their nanosize and abundance of atoms on their surfaces, inorganic NPs can exhibit properties such as magnetism, conductivity, radioactivity, and light (heat) responsiveness (Auffan et al., 2009). Based on these properties, inorganic NPs are increasingly used in the field of biomedicine for applications such as therapy, diagnosis, biosensors, and material component modules (Ni et al., 2017; Hess et al., 2019; Luther et al., 2020; Mitchell et al., 2021). The most commonly used inorganic materials include pure metals (e.g., gold and silver), metal oxides (e.g., mesoporous silica and γ-Fe_2_O_3_/Fe_3_O_4_), semiconductor materials, and calcium phosphate.

**FIGURE 4:**
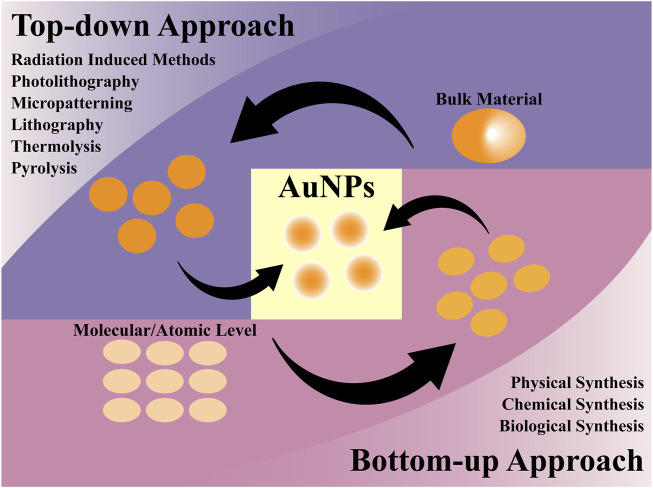
Method of preparing AuNPs.

Compared to other inorganic NPs, noble metal (e.g., Au, Ag, Pt, Hg, and Cu) NPs are gaining increasing attention from researchers (Ramalingam et al., 2014). Among them, gold NPs are known to be the most stable NPs. They also possess tunable optical properties, which are determined by the surface plasmon resonance effect, involving the oscillation and interaction of electrons between surface negative and positive charges (Ramalingam, 2019). As shown in Figure 4, gold NPs can be synthesized using top-down and bottom-up approaches. However, these methods often face challenges such as the use of chemically toxic reagents with safety risks, complex preparation processes, and the need for improved functionality and biocompatibility (da Silva et al., 2020). For example, common surfactants like cetyltrimethylammonium bromide and reducing agents such as hydrazine hydrate and sodium borohydride, due to their explicit toxicity, must be removed or encapsulated with biocompatible shells during the preparation process (LunáCheung et al., 2012; Kumar et al., 2019). To avoid toxicity risks and explore diverse biomedical applications, the green synthesis of gold NPs based on specific natural compounds is considered an ideal alternative method for improving the preparation process and enhancing the functionality of the final materials. These natural bioactive components also possess inherent pharmacological properties. Some active ingredients, such as CUR (Matur et al., 2020), resveratrol (Wang et al., 2017), and epigallocatechin gallate (EGCG) (Wu et al., 2018) have received increasing attention due to their unique structures and physicochemical characteristics that can confer special functions to materials. For instance, Yao et al. (2022) selected the tetracyclic triterpenoid compound ginsenoside Rh2 from *Panax ginseng* C.A. Meyer as a reducing agent and stabilizer to react with HAuCl_4_, resulting in the synthesis of Au@ginsenoside Rh2 NPs. These NPs inherited the excellent anticancer properties of ginsenoside Rh2 and improved its poor water solubility.

##### 3.2.3.3 Bio-NPs

Currently, nanocarriers can be broadly classified into two categories: artificial nanocarriers and natural nanocarriers. Artificial nanocarriers, represented by inorganic NPs, have limitations in drug loading capacity and inevitable systemic toxicity, which to restrict their application as delivery platforms (Chen et al., 2022). The safety concerns associated with using artificially synthesized materials for drug delivery have accelerated the research and application of cell-derived nanovesicles (CDNs). CDNs include naturally secreted extracellular vesicles (e.g., exosomes and microvesicles), stimulus-induced nanovesicles, and lipid-based nanovesicles (Liu et al., 2021). CDNs are typically isolated and purified from culture media and various biological fluids, or they can be produced or modified from various cells, bacteria, fungi, or even whole plants. Currently, there are three main methods for preparing CDNs. The first method involves the separation and purification of naturally secreted CDNs using techniques such as differential ultracentrifugation (DUC), density gradient ultracentrifugation (DGUC), and ultrafiltration (UF) (Li et al., 2017). The second method involves the application of exogenous stimuli to host cells to enhance the biogenesis of CDNs, which is a feasible approach for increasing CDN production while maintaining the major characteristics of the cell membrane (Zou et al., 2019). The last method involves the extraction of bio-lipids from cells and the reconstruction of CDNs *in vitro* (Yang et al., 2018).

As particles that exist naturally in the environment, CDNs possess almost all the advantages of artificial nanocarriers, and they also exhibit biocompatibility and biosafety (Johnsen et al., 2018). Through optimized separation, detailed characterization, and appropriate functionalization, CDNs have been successfully prepared for delivering plant chemicals. In the field of TCM, CDNs have been studied for delivering active ingredients such as CUR, RES, QUE, triptolide, and BBR. Table 2 summarizes the reports on the delivery of herbal ingredients using CDNs.

**TABLE 2:** Research on CDNs as delivery carriers for active ingredients of TCM.

Drug	CDNs source	Preparation	Application	Ref.
Quercetin	Plasma exosome	Isolation	Alzheimer’s Disease	Qi et al. (2020b)
Icariin	Fetal Bovine Serum	Isolation	Bone Loss Disease	Dong et al. (2021)
Triptolide	ID8 Cell	Isolation	Ovarian Cancer	He et al. (2021)
	SKOV3 Cells	Isolation	Ovarian Cancer	Liu et al. (2019a)
Paclitaxel	Milk	Isolation	Lung Cancer	Aqil et al. (2016)
	Dendritic Cells	Isolation	Breast Cancer	Wan et al. (2018)
	RAW 264.7 Cell	Isolation	Lung Cancer	Kim et al. (2016)
Camptothecin	Mesenchymal Stem Cell	Isolation	Tendon Injury	Li et al. (2020a)
Berberine	M2 macrophage	Isolation	Spinal Cord Injury	Gao et al. (2021)
Curcumin	Milk	Isolation	Cervical Cancer	Aqil et al. (2017)
	Endothelial Cell	Isolation	Cerebral Diseases	Kalani et al. (2014)
	Raw 264.7 Cell	Isolation	Glioma	Jia et al. (2018)
Resveratrol	RBC Membranes	Bio-fabrication	Alzheimer’s Disease	Han et al. (2020)

The application of CDNs for delivering active ingredients also faces some challenges. The primary issue is the limited efficiency in obtaining CDNs, especially in regard to large-scale and highly selective separation and preparation of CDNs from complex media, which requires further research. Additionally, the functional modifications carried out on CDNs to achieve therapeutic goals may compromise their structural integrity, reduce drug loading capacity, and alter the *in vivo* distribution of the drugs (Chen et al., 2022). Therefore, the selection and optimization of CDN-based drug delivery systems require further consideration and improvement.

#### 3.2.4 Dendrimers

Dendrimers are large molecules with a dendritic structure, that consist of oligomers that are repeatedly and linearly linked by branching units. As the number of polymerization generations increases, the degree of branching of the molecules continues to expand, eventually leading to the formation of a closed three-dimensional spherical structure (Abbasi et al., 2014). The number of branching points (also known as focal points) from the central core to the surface is referred to as “generation” (Tomalia, 2005). For instance, dendrimers with 5 branching points are called the “fifth generation” and are denoted as “G5-dendrimer”. Therefore, the fifth generation PAMAM dendrimer is referred to as G5-PAMAM.

Dendrimers possess controllable physicochemical properties, enriched active functional groups, and internal cavity structures, making them promising drug delivery carriers (Madaan et al., 2014). There are two approaches for dendrimer-based drug delivery. One is through non-covalent interactions, where the drug molecules are encapsulated within the internal cavities of dendrimers, providing protection against metabolic processes and enhancing the bioavailability of the drugs. The other approach involves covalent interactions, where the drug molecules are covalently linked to the dendrimers using cleavable functional groups such as esters and amines, enabling effective drug release and controlled release of the drugs (Chis et al., 2020).

Compared to more mature technologies such as liposomes, NPs, and micelles, dendrimers have a relatively late start in the field of drug delivery. Currently, one of the most successful companies utilizing dendrimers as drug delivery platforms is Starpharma from Australia. Their DEP^®^ platform has advanced multiple drugs to clinical stages (Kelly et al., 2020). In the field of TCM, there have been reports on the use of dendrimers for the delivery of active ingredients such as MT (Alibolandi et al., 2017), PTX (Bhatt et al., 2019), BBR(Yadav et al., 2023), QUE (Madaan et al., 2016), ellagic acid (Priyadarshi et al., 2021), RES (Pentek et al., 2017), and CUR (Gamage et al., 2016).

### 3.3 Carrier-free nanomedicines

Currently, the development of nanocrystallization of single active ingredients has to some extent overlooked the compatibility of TCM formulas. This is because clinically used TCM contain various components with different physicochemical properties. Therefore, it is necessary to explore multi-component nanomedicines. The theory of TCM compatibility refers to the combination of various herbs, reflecting the synergistic effects of different components. Utilizing multiple active ingredients through nanotechnology is an effective strategy. Some structurally ideal active ingredients can be utilized as carriers, while some active ingredients can act simultaneously as carriers and drugs, fully exploiting their physicochemical properties and pharmacological actions. Various nanostructures formed by self-assembly of active ingredients of TCM are referred to as carrier-free nanomedicines (Li L. et al., 2020).

#### 3.3.1 Self-assembled nanomedicines

**FIGURE 5:**
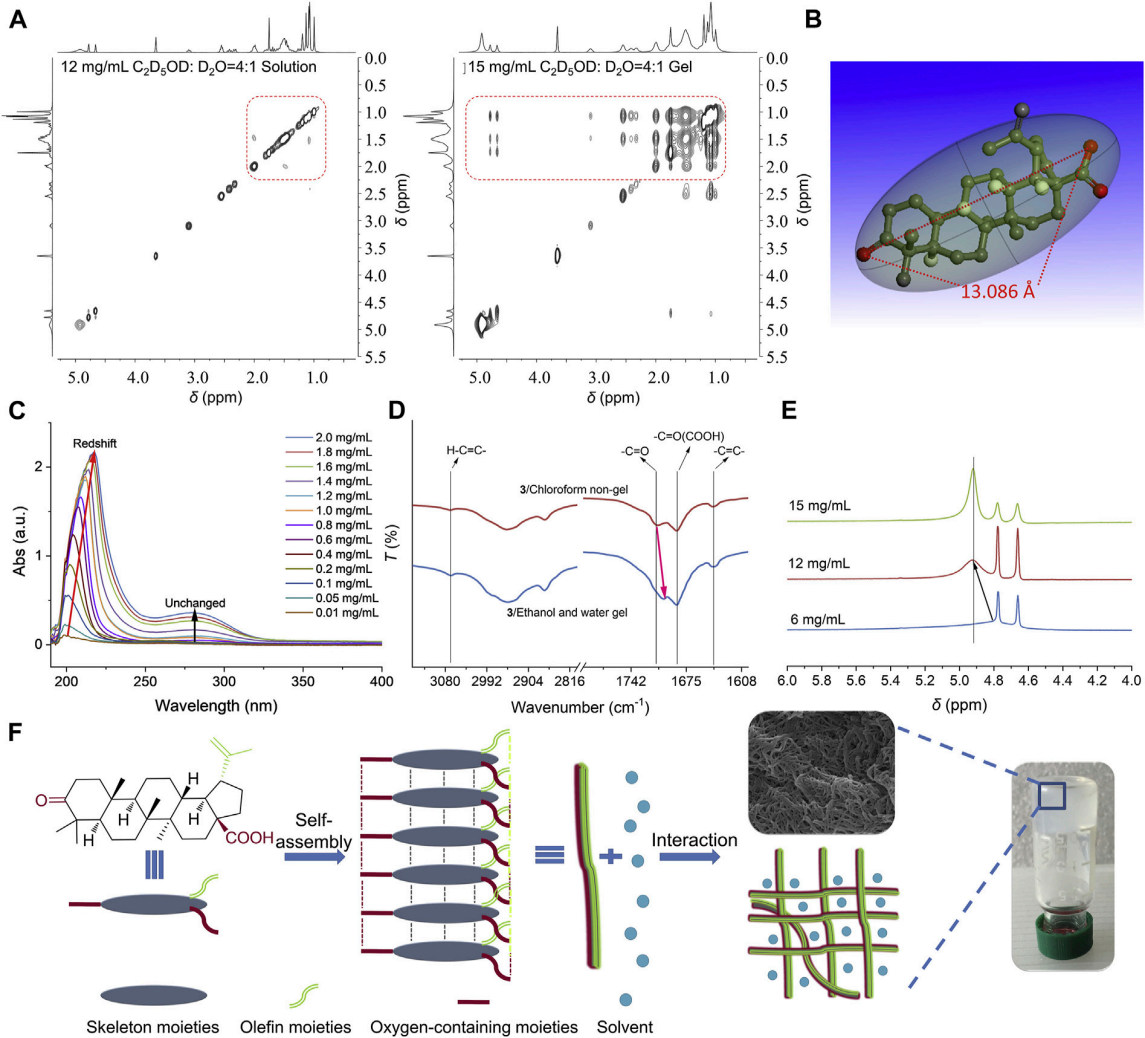
Formation of injectable NPG scaffold. **(A)** 2D NOESY spectra of compound 3 in mixed solvent of deuterated ethanol and deuterated water (4:1) at different concentrations. **(B)** Molecular length of compound 3. **(C)** UV spectra of compound 3 in ethanol/water mixed solvent (1:1) at different concentrations. **(D)** IR spectra of compound 3 obtained from non-gel and gel. **(E)** 1H NMR spectra of compound 3 in mixed solvent of deuterated ethanol and deuterated water (4:1) at different concentrations. **(F)** A possible self-assembly formation process of NPG. Reprinted from Acta Pharmaceutica Sinica B, K. Zhi, J. Wang, H. Zhao, X. Yang, Self-assembled small molecule natural product gel for drug delivery: a breakthrough in new application of small molecule natural products, 913-927, Copyright (2020), with permission from Elsevier [OR APPLICABLE SOCIETY COPYRIGHT OWNER].

Carrier-free self-assembled nanomedicines of TCM refer to the formation of stable and specific structures through non-covalent interactions such as hydrogen bonding, van der Waals forces, π-π stacking, electrostatic interactions, and coordination bonds between active ingredients of TCM(Tian et al., 2020). Studies have found that the structural diversity of herbal ingredients enables their self-assembly capabilities, allowing them to assemble with other molecules through non-electrostatic interactions. The preparation of self-assembled nanomedicines is simple and allows for high drug loading, while achieving highly stable drug delivery without the use of carriers (Figure 5) (Zhi et al., 2020). Currently, common ingredients of TCM with self-assembly properties include terpenoids, glycosides (Mao et al., 2022), and quinones (Wu et al., 2022). These natural molecules can self-assemble at the interface of different solvents to form carrier-free nanomedicines.

Ginsenosides, the main active ingredients of *P. ginseng* C.A., belong to triterpenoid saponins. In order to avoid potential issues associated with poor biocompatibility, low drug loading capacity, and unpredictable side effects associated with drug carriers, Tan et al. (2022) utilized the intermolecular recognition of different ginsenoside monomers to achieve self-assembled carrier-free ginsenosides nano-micelles (GSN). These self-assembled micelles exhibited a lamellar structure with a uniform particle size distribution. The molecular interactions between ginsenosides were preliminarily studied using Discovery Studio 4.0 (DS 4.0). The results demonstrated that the formation of GSN was driven by alkyl-alkyl interactions and hydrogen bonding. Additionally, GSN effectively inhibited tumor cell adhesion activity and the expression of intercellular adhesion molecule-1 (ICAM-1). Importantly, in an *in vivo* H22 mouse artificial lung metastasis model, the self-assembled system significantly inhibited tumor metastasis. These results suggest that this carrier-free nanomedicine has potential for the treatment of tumor metastasis.

Rhein is an anthraquinone derivative and is present in *Rheum palmatum* L. In the absence of carrier materials, Wu et al. (2021) utilized hydrogen bonding and π-π stacking interactions as driving forces to self-assemble rhein and DOX into a mitochondria-targeting nanogel. This nanogel achieved 100% drug loading and not only enabled sustained controlled release but also overcame the drawbacks associated with the use of free DOX and free rhein, such as high toxicity, poor target specificity, low solubility, and low bioavailability. In the tumor environment, the rhein-DOX nanogel was taken up by HepG2 cells and delivered to the mitochondria. Subsequently, rhein and DOX were released from the fibrous structure. The rhein-DOX nanogel significantly increased intracellular reactive oxygen species (ROS) levels, decreased mitochondrial membrane potential (MMP), and further induced cell apoptosis. These results demonstrated the synergistic effect of rhein and DOX in the treatment of liver cancer.

Ursolic acid is a pentacyclic triterpenoid compound. Fan et al. (2018) designed a carrier-free nanomedicine based on the self-assembly of ursolic acid molecules. This process relies on hydrogen bonding and hydrophobic interactions between ursolic acid molecules, resulting in stable NPs with a particle size of 100–200 nm and a high drug loading capacity of up to 60%. Compared to free ursolic acid, these nanomaterials significantly inhibit cancer cell proliferation and induce apoptosis. In *vivo* studies, the nanomaterial significantly inhibited tumor growth and protected the liver in A549 xenograft mouse models. This carrier-free nanomedicine platform represents a strategy to enhance the anticancer effects of poorly soluble drugs.

#### 3.3.2 Pharmaceutical cocrystals

Pharmaceutical cocrystals refer to multicomponent molecular crystals formed by two or more drug molecules through hydrogen bonding or other non-covalent interactions, with at least one molecule being an active pharmaceutical ingredient (API) and the other being a co-former (Kuminek et al., 2016; Gu et al., 2022). Traditional co-formers are often composed of safe pharmaceutical excipients, but there are now drug cocrystals composed solely of active drug substances that have been used in clinical applications. These drug cocrystals retain the advantages of individual components while exhibiting synergistic effects in terms of pharmacological activity. For example, the sacubitril valsartan sodium cocrystal has been used in the clinical treatment of heart failure (McCormack, 2016).

In the field of TCM, non-dissociating and weakly dissociating active ingredients, such as flavonoids, alkaloids, terpenoids, and polyphenols, can form cocrystal structures through intermolecular interactions like hydrogen bonding (Heng et al., 2022). In particular, due to the competitive hydrogen bonding sites within the molecular framework of flavonoids, they readily form cocrystals with co-formers that also contain hydrogen bond acceptors and donors. Currently, a series of cocrystals formed between flavonoid compounds and excipients, such as caffeine, isoniazid, nicotinamide, acetamide, betaine, and theophylline have emerged. These cocrystals exhibit good solubility, dissolution, and oral bioavailability. For instance, Luo et al. (2019) synthesized a cocrystal of luteolin with isoniazid and caffeine using liquid-assisted grinding. The solubility of the luteolin-isoniazid cocrystal was 112.3 μg/mL, approximately three times greater than that of free luteolin. According to the pharmacokinetic analysis, compared with those of free luteolin, the AUC_0-∞_ of the luteolin-isoniazid cocrystal and luteolin-caffeine cocrystal were 2.7-fold and 1.4-fold greater, respectively.

#### 3.3.3 Pickering emulsion

Pickering emulsion is a type of emulsion in which solid particles act as stabilizers and adsorb onto the surface of liquid droplets. This structural uniqueness endows the material with excellent stability, biocompatibility and environmental friendliness (Ni et al., 2022). The use of Pickering emulsion can significantly improve the oral bioavailability of poorly soluble drugs such as CUR, silybin, puerarin, and rutin (Tai et al., 2020). In terms of drug delivery systems, lipophilic components can be loaded into the oil phase, hydrophilic drugs can be loaded into the aqueous phase, and amphiphilic drugs can be loaded at the oil-water interface. Additionally, Pickering emulsion can be used as precursors for preparing other dosage forms or carriers, such as nano-composite materials, magnetic solid microspheres, and hollow microcapsules (Nypelö et al., 2014).

#### 3.3.4 Nanosized aggregates in decoction

Decoction is the main form of clinical application of TCM(Weng et al., 2019). The decoction with water as the solvent contains complex active ingredients, and it is speculated that hydrophobic compounds may be modified to increase their solubility (Kim and Park, 2017). Due to the encapsulation of hydrophobic components by amphiphilic polysaccharides or proteins, as well as the interaction between acidic and alkaline compounds, new composites may be formed during the decoction process of TCM(Zhou et al., 2019).

Liu et al. successfully isolated and characterized nanosized aggregates from Bai-Hu Tang, which exhibit excellent performance in antipyretic properties (Lü et al., 2018). Zhuang et al. found that the nanosized aggregates of Xue-Fu-Zhu-Yu Tang during boiling are closely related to its protective effect on cardiovascular system (Zhuang et al., 2008). In addition, Zhou et al. extracted colloidal NPs from Ma-Xing-Shi-Gan Tang, which were formed by hydrophobic or ionic interactions between amphiphilic molecules such as ephedrine and pseudoephedrine (Zhou et al., 2014). In summary, substances such as nanosized aggregates have played a crucial role in TCM decoctions, which has been proven through nanotechnology. This discovery not only provides ideas for understanding the mechanism of TCM, but also provides valuable reference for the development of innovative dosage forms (Zheng et al., 2022).

## 4 The safety of nano-TCM

Nano-based drug delivery systems hold promise for traversing biological barriers, including cell membranes and even the BBB(Cox et al., 2018; Tosi et al., 2020). However, concerns regarding their potential toxicity are also increasing. The toxicity mechanism of nano-TCM is relatively complex, which is not only related to the toxic components contained in the drug, but also to factors such as drug metabolism and elimination in the body. The advantages of improved solubility and enhanced targeting provided by nanomedicines can reduce or eliminate the toxic effects of active ingredients of TCM. TP has remarkable efficacy in anti-tumor and anti-autoimmune effects, but it is accompanied by serious adverse effects, such as toxic effects on multiple organs (liver, kidney, heart and reproductive system) (Ma et al., 2015; Wang et al., 2019). In addition, clinical applications are limited due to its poor water solubility. To overcome these problems, researchers developed a transferrin-modified TP liposome (TF-TP@LIP). This modification significantly enhances the liposome’s ability to target tumors and reduces the accumulation of the drug in non-target tissues and organs, thereby reducing drug toxicity and adverse effects (Zhao et al., 2023).

In addition, the interaction between active ingredients of TCM can also produce a detoxification effect. Licorice, the dried root and rhizome of *Glycyrrhiza uralensis* Fisch., *Glycyrrhiza inflata* Bat., or *Glycyrrhiza glabra* L., is an “essential herbal medicine” in TCM. It can reduce toxicity and improve efficacy in the combination application of certain herbs. Jiang et al. conducted a study on 124 bibliographies published from 1976 to 2019 and found that the interaction between licorice and toxic compounds, as well as the influence of licorice on the metabolism of toxic compounds, are the main mechanisms by which licorice plays a role in TCM formulas (Jiang et al., 2020). Euodiae Fructus (EF) is a commonly used herb with mild toxicity in clinic. Zhang et al. found that licorice processing can significantly reduce the hepatotoxicity of EF. The detoxification mechanism may be related to the antagonistic effect of licorice on toxic components (Zhang et al., 2021).

## 5 Challenges and opportunities

The discovery of famous drugs such as ART and BBR usually follows the concepts and strategies of Western medicine, which involve developing new drugs using isolated single natural products (You et al., 2022). This is one way in which TCM has contributed to the development of global medicine. However, there are obvious limitations to this research approach as it lacks the guidance of TCM theory. TCM involves the individual regulation of multiple components and targets, allowing the body to transition from an abnormal state to a normal state. This characteristic makes it difficult to replicate and conduct large-scale clinical trials, thus making it challenging to obtain statistically significant results (You et al., 2022). Through genomics, transcriptomics, proteomics, metabolomics and combined omics analysis, we are able to gain a more comprehensive understanding of the interactions between TCM and biological system. Due to the complexity and multi-component nature of TCM prescriptions, the application of such research techniques can not only provide a deeper understanding of drug mechanisms but also enhance the knowledge of TCM principles, so that the embedded ancient wisdom can be reinterpreted and utilized through the lens of modern science.

## 6 Conclusion

Nanotechnology, as a field with tremendous potential, has brought new momentum and confidence to the modernization of TCM. On the one hand, relying on advantages such as improving bioavailability, achieving controllable release, and enhancing targeted effects, developing single active ingredients into nanomedicines is considered an innovative strategy for the development of new drugs. On the other hand, interpreting the mechanism of compound prescriptions of TCM is crucial for the modernization of TCM. Exploring the substance basis of TCM formulas from the perspective of nanotechnology can provide strong support for elucidating the scientific intension of TCM formulas. In addition, exploring the physiological and biochemical responses of nano-TCM in human body with the help of omics technology is of great value in elucidating the pharmacological mechanism and targets of herbal medicines. It is worth emphasizing that the continuous integration of TCM with modern scientific principles and technologies will continue to serve the promotion of human health.

## Figures and Tables

**Table:** 

ART	artemisinin
AT	acyltransferase
BBR	berberine
BBB	blood-brain barrier
CDNs	cell-derived nanovesicles
CPT	camptothecin
CYP450	cytochrome P450
DOX	doxorubicin
DMAPP	dimethylallyl pyrophosphate
EPH	ephedrine
EGCG	epigallocatechin gallate
EE	encapsulation efficiency
FDA	the U.S. Food and Drug Administration
FA	folic acid
GSN	ginsenosides nano-micelles
GMA	glycidyl methacrylate
HDF	human dermal fibroblasts
HEMA	2-hydroxyethyl methacrylate
LF	lactoferrin
LECs	lipid-based nanocarriers
MT	matrine
NPs	nanoparticles
PTX	paclitaxel
PFH	perfluorohexane
PEG	polyethylene glycol
PLA	polylactic acid
PLGA	poly (lactic-co-glycolic acid)
PCL	poly-ε-caprolactone
PAMAM	poly (amidoamine)
PLL	poly (L-lysine)
PEI	polyethylenimine
PPI	poly (propylene imine)
QUE	quercetin
RES	resveratrol
ROS	reactive oxygen species
SCF	supercritical fluid
TPS	terpene synthase
TCM	Traditional Chinese medicine
TF	transferrin
TP	triptolide
UGT	UDP-glycosyltransferase
